# Periostin as a Tissue and Urinary Biomarker of Renal Injury in Type 2 Diabetes Mellitus

**DOI:** 10.1371/journal.pone.0124055

**Published:** 2015-04-17

**Authors:** Bancha Satirapoj, Surat Tassanasorn, Mongkon Charoenpitakchai, Ouppatham Supasyndh

**Affiliations:** 1 Division of Nephrology, Department of Medicine, Phramongkutklao Hospital and College of Medicine, Bangkok, Thailand; 2 Department of Pathology, Phramongkutklao Hospital and College of Medicine, Bangkok, Thailand; University of Louisville, UNITED STATES

## Abstract

**Background:**

Improving the early detection of diabetic nephropathy remains a great challenge in disease management. Periostin is a marker of renal tubular injury and related to progressive kidney injury in animal models of chronic kidney disease. The clinical implications of urinary periostin activities in patients with type 2 diabetes have not been evaluated.

**Methods:**

Urine samples were obtained from 30 healthy volunteers and 328 type 2 diabetic patients with normoalbuminuria (n=114), microalbuminuria (n=100) and macroalbuminuria (n=114). The excretion levels of urinary periostin were quantified with enzyme-linked immunosorbent assay. Immunohistochemical periostin expression was determined in kidney tissues from overt diabetic nephropathy.

**Results:**

Increased periostin expression in glomeruli and tubular epithelium in diabetic renal pathology was observed. Urinary periostin levels were significantly elevated in the patients of the normoalbuminuria [3.06 (IQR: 1.12, 6.77) ng/mgCr], microalbuminuria [8.71 (IQR: 5.09, 19.29) ng/mgCr] and macroalbuminuria [13.58 (IQR: 3.99, 16.19) ng/mgCr] compared with healthy controls [1.15 (IQR: 0.60, 1.63) ng/mgCr] (P<0.01).Increased urine periostin level significantly correlated with aging, high albuminuria and decline of GFR. Urine periostin ELISA also demonstrated high performance for the diagnosis of established normoalbuminuric, microalbuminuric and macroalbuminuric type 2 diabetes (AUC 0.78 (95%CI, 0.71 to 0.86), 0.99 (95%CI, 0.98 to 1.00) and 0.95 (95%CI, 0.91 to 0.98), respectively).

**Conclusion:**

The study indicates that increased urine periostin levels can be detected in patients with type 2 diabetes before the onset of significant albuminuria. Urinary periostin is an associated renal derangement in patients with established diabetic nephropathy and it may be used as an early marker of diabetic renal injury.

## Introduction

Patients with diabetic nephropathy have a higher risk of mortality, mostly from cardiovascular complications, than diabetic patients without nephropathy [1]. Nephropathy in diabetic patients is characterized by functional as well as structural abnormalities [2]. In additional to diabetic glomerular pathology, the disease progresses in the tubulointerstitial compartment causing the expansion of tubular basement membranes, tubular atrophy, interstitial fibrosis, and arteriosclerosis. Tubulointerstitial damage is one of important factors in the pathophysiology and progression of nephropathy [3]. Moreover, it has been proposed that tubular injury could precede glomerular injury in diabetic nephropathy [4,5], which may explain the early appearance of an increase in several urinary biomarker excretion compared with albumin. Importantly, one third of patients with diabetic nephropathy lost renal function even during normal albuminuria or without any marker of glomerular injury [6]. It further focuses on tubulointerstitial injury biomarkers that could be used to monitor the progression of diabetic nephropathy and facilitate its diagnosis and treatment.

Periostin, originally identified in osteoblasts, functions as a cell adhesion molecule for preosteoblasts and is not observed in adult kidneys under normal conditions [7]. We have previously demonstrated that periostin was prominently expressed in tubulointerstitial areas during renal injury and periostin in the urine was a measure of the loss of renal tubular cells that had adopted a mesenchymal phenotype in response to diverse renal injuries [8]. Its histopathologic expression patterns in the kidney in situ suggest that periostin participates in the pathogenesis of renal disease in response to transforming growth factor-beta (TGF-beta) and that blocking periostin expression protected animals from renal injury [9]. Recent studies have demonstrated that periostin was excreted in the urine of experimental animal models of chronic kidney disease (CKD) and patients with various kidney diseases including chronic allograft nephropathy (CAN) [10–12]. However, no quantitative information exists on the relationship between urinary periostin and clinical staging of diabetic nephropathy. The aim of the present study was to investigate the determinants of urinary periostin and their associations with severe diseases of diabetic nephropathy.

## Materials and Methods

### Ethics statement and Participants

The study protocol was approved by the human subjects institutional review board of the Royal Thai Army Medical Department, Bangkok, Thailand. We obtained written informed consent from each patient. Type 2 diabetes Thai subjects were recruited from the Outpatient Nephrology Clinic. Inclusion criteria of the study included age, 18 years or older and type 2 diabetes patients. Patients with active urinary tract infection, renal disease other than diabetic nephropathy, neoplastic disorders, severe liver disease, active or chronic infection or inflammatory disorders, pregnancy or a recent history of acute myocardial infarction, stroke, or occlusive peripheral vascular disease were excluded from this study. Medical history was taken including current smoking and alcohol consumption, defined as the consumption of up to one drink per day for women and up to two drinks per day for men. In addition, systolic and diastolic blood pressure, body weight, body mass index, routine laboratory data including blood urea nitrogen (BUN), serum creatinine, urinary albumin creatinine ratio (UACR) and estimated GFR from calibrated serum creatinine with the 2009 CKD-EPI creatinine equation [13] were recorded.

### Sample collection

Early morning urine samples of healthy subjects without type 2 diabetes (n = 30) and every possible patient with type 2 diabetes with various stages of normoalbuminuria (urine albumin creatinine ratio (UACR) <30 mg albumin/g creatinine, N = 114), microalbuminuria (UACR 30–300 mg albumin/g creatinine, N = 100) and macroalbuminuria (UACR >300 mg albumin/g creatinine and/or persistent proteinuria, N = 114) were obtained. Spot urinary albumin and creatinine concentrations were measured, and expressed as the UACR. Urine samples were centrifuged for 10 minutes at 3,000 rpm to remove particulate matter and stored at -80°C until analysis.

### Immunohistochemical study

Renal biopsies from patients (3 normal kidneys, 3 with overt diabetic nephropathy) were retrospectively collected for periostin immunostaining. In brief, paraffin-embedded tissue sections were deparaffinized, rehydrated and incubated in 3% hydrogen peroxide (to block endogenous peroxidases). Antigen retrieval was performed in HCl (pH 0.9) heated by microwaving for 10 minutes. Staining was performed at 4°C overnight with antibodies to polyclonal periostin (BioVendor, Candler, NC, 1:250), followed by incubation with dextran polymer conjugated with horseradish peroxidase and affinity-isolated immunoglobulin for 30 minutes at room temperature. Immunoreactive proteins were visualized with a 3-amino-9-ethylcarbazole-containing peroxydase kit (Dako) and counterstained with hematoxylin. For negative controls, the primary antibody was replaced by an equal concentration of rabbit or mouse IgG.

### Urinary periostin measurement

A total of 96-well microplates were coated overnight with 1 μg/mL (0.1 μg per well) of anti-periostin antibody (R&D Systems, Minneapolis, MN). Plates were washed three times with 0.05% Tween 20 in PBS then blocked with reagent diluent for at least one hour. A total of 100 μL of all standards and patient samples was added to the 96-well plate and incubated for 2 hours. After 1-hour incubation with rabbit polyclonal anti-periostin antibody (Abcam, Cambridge, UK, 1:1000), 20 minute incubation with dextran polymer conjugated with horseradish peroxidase, and 20 minute incubation with substrate solution, stop solution was added to each well. Periostin absorbance was calculated by measuring at 450 nm, correcting for plate artifact at 570 nm using a log-transformed standard curve. Intra-patient variability of urine periostin was less than 12.5%.

### Statistical analysis

Results were expressed as means ± SD and median with interquartile (IQR) for continuous variables or as a percentage in categorical variables. Statistical analysis was performed using SPSS, version 15. Either the two-sample *t* test or Mann-Whitney rank sum test was used for continuous variables. For multiple comparisons, ANOVA was used followed by the least significance difference test. Moreover, Spearman’s correlation coefficients were used as appropriate to test correlations between urine angiotensinogen and other variables. Multiple regression was used to detect urine periostin and the relevancy of each parameter. Finally, receiver operating characteristics (ROC) analysis was used to calculate the area under the curve (AUC) for periostin and to find the best cut-off values to identify diabetic nephropathy. Results with p<0.05 were considered statistically significant.

## Results

### Patient characteristics

Type 2 diabetes patients with normoalbuminuria (n = 114), microalbuminuria (n = 100) and macroalbuminuria (n = 114) and healthy volunteers (n = 30) were enrolled in the study. Table 1 and 2 summarize the baseline characteristics. All characteristics were similar among three groups of diabetic patients except that significantly increased systolic blood pressure was detected in the macroalbuminuria group (Table 1). Fasting plasma glucose and hemoglobinA1C were significantly higher in all groups of diabetic patients compared with the control. As expected, serum creatinine and UACR were significantly elevated in patients with advance stage of diabetic nephropathy compared with type 2 diabetes with normoalbuminuria and healthy controls. However, no significant difference of UACR was observed between normoalbuminuria and control groups (Table 2).

**Table 1:** Baseline clinical characteristics.

	Control (n = 30)	Normoalbuminuria (N = 114)	Microalbuminura (N = 100)	Macroalbuminura (N = 114)
Age (year)	42.33±5.05	64.61±8.95^a^	65.55±11.36^a^	65.53±10.81^a^
Male (%)	16 (53.33%)	62 (54.39%)	40 (40%)	63 (55.26%)
Duration of diabetes (year)	-	10.03±7.3	8.98±5.34	10.01±6.95
Hypertension (N, %)	-	106 (92.98%)	91 (91.92%)	110 (96.49%)
Using RAAS inhibitors (N, %)	-	72 (63.15%)	65 (65%)	69 (60.5%)
Dyslipidemia (N, %)	-	103 (90.35%)	93 (93.94%)	103 (90.35%)
Ischemic heart disease (N, %)	-	9 (7.89%)	12 (12.12%)	9 (7.89%)
Smoking (N, %)	-	22 (20.95%)	23 (23%)	20 (17.54%)
Alcoholic drinking (N, %)	-	26 (25.24%)	17 (18.89%)	24 (25.81%)
Body weight (kg)	67.88±12.01	71.27±16.11	66.65±13.91	68.95±14.01
Body-mass index (kg/m^2^)	26.85±4.03	27.55±5.13	25.8±4.22	26.82±4.72
Systolic blood pressure (mmHg)	126.38±12.71	131.90±16.71	131.37±16.95	139.49±16.70^a^,^b^
Diastolic blood pressure (mmHg)	79.58±11.14	74.92±9.97	72.94±11.38	75.88±10.65

Data are mean ± SD, median with interquartile range and percentages;

^a^P<0.05 versus normoalbuminuria,

^b^P<0.05 versus microalbuminuria, RAAS;

renin—angiotensin—aldosterone system

**Table 2:** Baseline laboratory profiles.

	Control (n = 30)	Normoalbuminuria (N = 114)	Microalbuminura (N = 100)	Macroalbuminura (N = 114)
FPG (mg/dL)	87.07±9.71	140.79±52.12^a^	149.39±53.51^a^	153.18±55.82^a^
HA1C (%)	5.46±0.35	7.22±1.47^a^	7.31±1.88^a^	7.94±2.68^a^
Total cholesterol (mg/dL)	211.47±34.25	154.82±24.89^a^	167.58±38.87 ^a^	179.79±57.59 ^a^ ^,^ ^b^
HDL (mg/dL)	56.85±18.01	54.2±17.11	50.55±13.32	49.63±14.58
LDL (mg/dL)	135.67±28.31	94.35±30.95^a^	101.2±34.11^a^	106.65±47.43^a^
TG (mg/dL)	136.67±86.99	117.52±48.07	134.41±59.57	165.72±132.79^b^
UACR (mg/g Cr)	4.45 (2.6, 5.5)	9.15 (4.3, 17.9)	65.5 (38.5, 120)^a^,^b^	458.7 (332.2, 507.7) ^a^,^b^,^c^
BUN (mg/dL)	11.2±3.25	17.57±12.69	17.74±9.47	28.74±18.25 ^a^,^b^,^c^
Serum creatinine (mg/dL)	0.78±0.18	1.1±0.43	1.11±0.59	2.16±1.74 ^a^, ^b^, ^c^
eGFR CKD-EPI (mL/min/1.73m^2^)	100.08±12.98	69.58±22.52^a^	69.94±28.32^a^	45.96±29.43^a^,^b^, ^c^

Data are mean ± SD, median with interquartile range and percentages;

^a^P<0.05 versus controls,

^b^P<0.05 versus normoalbuminuria,

^c^P<0.05 versus microralbuminuria. BUN, blood urea nitrogen, eGFR; estimated glomerular filtration rate, FPG; fasting plasma glucose, HDL; high density lipoprotein, HbA1C; hemoglobinA1C, LDL; low density lipoprotein, TG: triglycerides, UACR; urine albumin creatinine ratio

### Enhanced renal periostin expression in diabetic nephropathy

**Fig 1:**
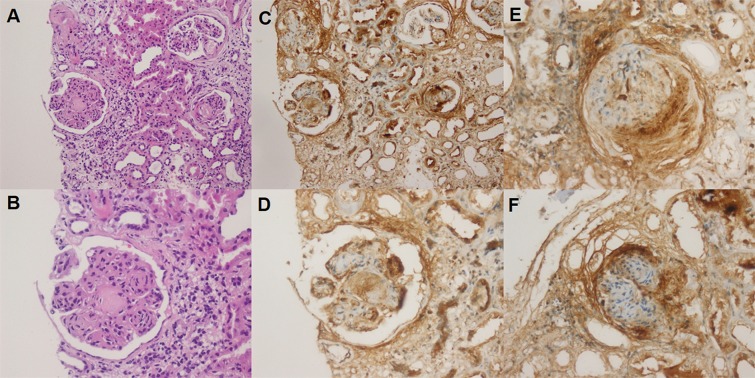
Renal periostin immunostaining in patients with diabetic nephropathy. Tissue sections were immunostained with a polyclonal rabbit anti-periostin antibody. Positive staining for periostin protein is shown in brown. All sections were counterstained with hematoxylin. Kidney tissues from patients with diabetic nephropathy display nodular glomerulosclerosis with arteriolar hyalinosis, interstitial fibrosis and tubular atrophy (A, B) and display diffuse periostin immunopositivity in glomerular and tubular compartments (C). Periostin staining was observed in the nodular sclerosis glomerulus with periglomerular and mesangial areas and also periostin staining in both atrophic and non-atrophic tubular epithelium (D). Periostin staining was found in the area of periglomerular fibrosis and collagen forming Bowman’s capsule within the ischemic type-change glomerulus (E). Global glomerulosclerosis showed periglomerular staining with periostin immunohistochemistry (F) (Original magnification: 400×).

Several kidney tissues from type 2 diabetic patients and controls were randomly collected for periostin immunostaining. Representative images of periostin immunostaining in patients with advanced diabetic nephropathy are shown in Fig 1. Representative sections of kidneys in the diabetic patients showed nodular glomerulosclerosis with arteriolar hyalinosis, interstitial fibrosis and tubular atrophy. In addition, they showed diffuse periostin immunopositivity in nodular sclerosis glomerulus and areas of periglomerular fibrosis and collagen forming Bowman’s capsule within the ischemic type-change glomerulus. Periostin also presented tubular cytoplasmic staining at both atrophic and nonatrophic tubular epithelium. In contrast, periostin was not detected in cortical control kidneys. Therefore, immunohistochemical analysis confirmed that increased periostin expression in pathologic glomeruli and tubules was observed after established diagnosis of diabetic nephropathy.

### Increased urinary periostin in type 2 diabetes

**Fig 2:**
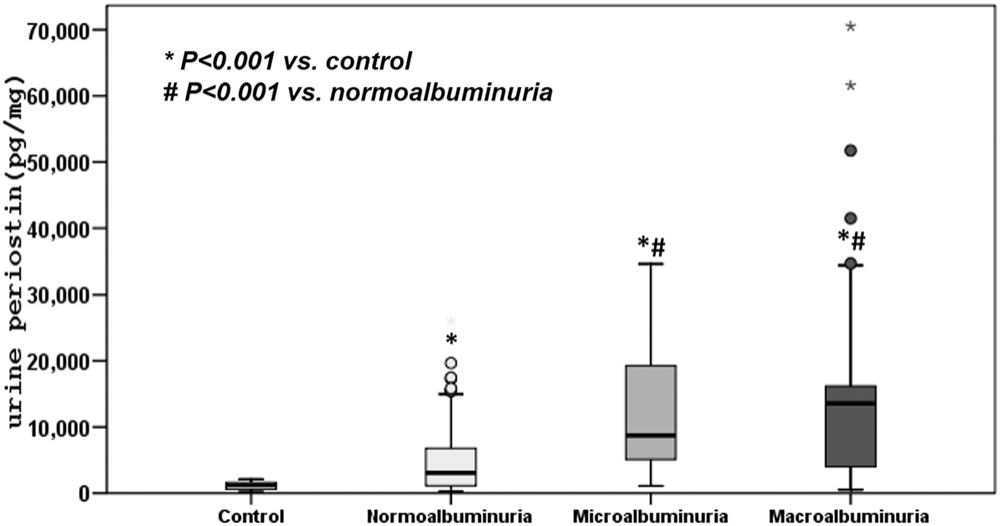
Urine periostin levels in patients with diabetic nephropathy and in healthy controls. Significant differences were observed between the urine periostin values in the each stage of diabetic nephropathy (P<0.001) compared with healthy subjects. Data are presented as the median with interquartile range. Open and filled circles were defined as outlier data more than 1.5 times of upper quartile.

Fig 2 shows the urinary periostin levels in healthy volunteers and patients with type 2 diabetes. Urine periostin was measured by ELISA and corrected for urine creatinine. Median urine periostin levels in healthy controls [1.15 (IQR: 0.60, 1.63) ng/mgCr] was significantly less than in patients with normoalbuminuric type 2 diabetes [3.06 (IQR: 1.12, 6.77) ng/mgCr, p<0.001], microalbuminuric type 2 diabetes [8.71 (IQR: 5.09, 19.29) ng/mgCr, p<0.001] and macroalbuminuric type 2 diabetes [13.58 (IQR: 3.99, 16.19) ng/mgCr, p<0.001] (Fig 2)(S1 Dataset). In addition, significant differences were found between the urine periostin values in normoalbuminuric type 2 diabetes and other stages of diabetic nephropathy. The appearance of urine periostin in type 2 diabetes patients but not in healthy controls underscores its value as a potential biomarker for kidney injury in albuminuric and nonalbuminuric type 2 diabetes.

### Correlation with urinary periostin

To evaluate the importance of periostin as a marker of kidney disease progression, we performed regression analyses with the hallmarks of diabetic nephropathy severity as dependent variables. Using univariate analysis, urine periostin levels were positively correlated with significance for age, fasting plasma glucose, hemoglobinA1C, plasma cholesterol and LDL, but negatively correlated with estimated GFR (Table 3). After multiple regression analyses, urine periostin levels were positively correlated with increased albuminuria, advanced age, and decline of estimated GFR (Table 3). In general, the increase in urine albumin, and the decrease in estimated GFR represent severe or aggravated renal function in patients with diabetic nephropathy. These data are consistent with the hypothesis that urinary periostin levels were associated with exaggerated renal function in type 2 diabetes patients.

**Table 3:** Univariate and multivariate linear regression analyses between urine periostin levels and other parameters in patients with type 2 diabetes.

	Univariate		Multivariate		
	*R*	*P value*	*Beta*	*95%CI*	*P value*
Age (yr)	0.156	*0*.*003*	132.70	13.23 to 252.17	*0*.*030*
FPG (mg/dL)	0.275	*<0*.*001*	5.30	-16.47 to 27.07	*0*.*632*
HbA1C (%)	0.109	*0*.*046*	69.38	-452.12 to 590.89	*0*.*793*
Cholesterol (mg/dL)	-0.106	*0*.*047*	4.87	-46.24 to 55.97	*0*.*851*
LDL (mg/dl)	-0.120	*0*.*024*	-2.83	-55.41 to 49.76	*0*.*916*
UACR (mg/g)	0.558	*<0*.*001*	16.79	10.56 to 23.01	*<0*.*001*
GFR-CKD EPI (mL/min/1.73 m^2^)	-0.073	*0*.*017*	-80.62	-30.35 to -130.90	*0*.*002*

Independent variables in the multivariate linear regression model were chosen using a stepwise regression analysis where all significant variables listed in the univariate analysis were included. FPG; fasting plasma glucose, HbA1C; hemoglobinA1C, eGFR; estimated glomerular filtration rate, LDL; low density lipoprotein, UACR; urine albumin creatinine ratio

### Performance of urinary periostin in diagnosing diabetic nephropathy

The ROC analysis of urine periostin (ng/mgCr) in diagnosing each stage of diabetic nephropathy is illustrated in Table 4. The AUC to diagnose established normoalbuminuric, microalbuminuric and macroalbuminuric type 2 diabetes using urine periostin were 0.78 (95%CI, 0.71 to 0.86), 0.99 (95%CI, 0.98 to 1.00) and 0.95 (95%CI, 0.91 to 0.98), respectively. The cut-off levels were 1.01 ng/mgCr (sensitivity 80.7%, specificity 43.3%), 1.13 ng/mgCr (sensitivity 74.6%, specificity 50.0%), 1.39 ng/mgCr (sensitivity 71.1%, specificity 66.7%) and 1.47 ng/mgCr (sensitivity 69.3%, specificity 70.0%) to distinguish normoalbuminuric type 2 diabetes from healthy controls. In addition, the cut-off levels of urine periostin to diagnose microalbuminuric and macroalbuminuric type 2 diabetes are also presented in Table 4. Therefore, urine periostin ELISA demonstrated moderate to high sensitivity and specificity for diagnosing diabetic nephropathy.

**Table 4:** Performance of urinary periostin in diagnosing diabetic nephropathy.

Group	Cut off value	Sensitivity (%)	Specificity (%)	Accuracy (%)	Area	95% CI	
						Upper	Lower
Urine periostin (ng/mg Cr) (ng/mgCr)							
Normoalbuminuria	1.01	80.7%	43.3%	72.9%	0.780	0.706	0.855
	1.13	74.6%	50.0%	69.4%			
	1.39	71.1%	66.7%	70.1%			
	1.47	69.3%	70.0%	69.4%			
Microalbuminuria	1.15	99.0%	50.0%	79.2%	0.993	0.981	1.000
	1.58	99.0%	73.3%	84.0%			
	2.00	98.0%	86.7%	86.1%			
	2.04	98.0%	96.7%	88.2%			
Macroalbuminuria	1.15	93.9%	50.0%	84.7%	0.947	0.913	0.981
	1.39	93.0%	66.7%	87.5%			
	1.88	89.5%	83.3%	88.2%			
	2.21	88.6%	100.0%	91.0%			

## Discussion

The present study describes the rising urine excretion of periostin in type 2 diabetes patients. Urinary periostin levels correlated with albuminuria and inversely with estimated GFR. In urine periostin, ELISA demonstrated moderate to high sensitivity and specificity for diagnosing diabetic nephropathy. The following novel findings emerged: urinary periostin was increased in normoalbuminuric type 2 diabetes compared with healthy controls. Taken together, these data demonstrate that periostin is a likely marker of renal tubular injury and a promising renal tissue and urine biomarker for kidney injury in early stages of type 2 diabetic nephropathy. Moreover, the increase in urine periostin represents the severity of renal injury in patients with diabetic nephropathy.

Epithelial to mesenchymal transition (EMT) has been proposed to contribute to tubulointerstitial fibrosis and to the progression of diabetic nephropathy.[14,15] Increased expression of TGF-beta in renal tissue and TGF-beta induced transformation of epithelial cells to cells with mesenchymal phenotype during progressive diabetic nephropathy has also been recently reported [16,17]. De novo expression of periostin during renal injury might be common events during renal tissue remodeling in a manner analogous to its functions in other injured tissues.[18,19] Periostin can induce cellular dedifferentiation, extracellular matrix deposition and increased TGF-beta expression [20]. In addition, administering TGF-beta to renal epithelial cells increased periostin expression and promoted loss of the renal tubular epithelial phenotype [9]. Previous studies have demonstrated that periostin was expressed predominantly in renal tissues in animal models after progressive renal injury, and periostin induced differentiated renal tubular cells in the mesenchymal phenotype among the rat models with CKD after 5/6 nephrectomy [8]. These findings collectively suggest increased renal periostin is part of an underlying EMT mechanism involved in diabetic kidney injury.

Consistent with the initial findings, biopsies from patients with hypertensive nephropathy, various glomerulopathies and CAN demonstrated intense periostin expression predominantly in the region presenting tubular atrophy, interstitial fibrosis, tubular epithelial cells, periglomerular fibrosis and glomerular mesangium [10–12]. Our study also confirmed that periostin staining was noted predominantly in atrophic and nonatrophic tubular epithelium, nodular sclerosis glomerulus with periglomerular and mesangial area in the setting of advanced diabetic nephropathy. Therefore, expression of periostin during different types of renal injury and among patients with type 2 diabetes, further suggests that periostin may serve as a quantitative marker of renal tubular loss linked to common pathogenic process in diabetic nephropathy.

Diabetic nephropathy is characterized by progressive destruction of the glomerular, tubulointerstitium and the loss of functional nephrons, finally leading to CKD. Numerous relationships between tubulointerstitial changes and functional outcomes have been reported [21]. The degree of tubulointerstitial damage is thought to be a stronger predictor of disease progression than the severity of damage to the glomeruli [22–24]. Urinary biomarker data in human beings support the view that tubular injury takes part in the development of early diabetic nephropathy [3]. Sensitive markers of tubular injury have been identified in CKD cases. Periostin is undetected in the normal renal tubules, and is released when distal tubules are damaged. Periostin levels in renal tissue and urine were significantly higher among diabetic rats [8] and patients with glomerulopathies, hypertensive nephropathy and CAN [10–12]. It also showed enhanced renal periostin expression and its staining correlated negatively with renal function [10–12]. In addition, increased urine levels of periostin can be detected by enzymatic assay in kidney diseases that have been reported in a limited number of CKD patients including patients with diabetic nephropathy [8]. The results of our study showed, for the first time in the literature, that in type 2 diabetic patients with normoalbuminuria, urinary periostin levels were significantly higher than in controls and urinary periostin excretion correlated with the severity of nephropathy in patients with type 2 diabetes. Therefore, urinary periostin might prove to be a sensitive biomarker to detect renal tubular injury of incipient nephropathy.

Our results identified periostin in urine of patients with the progression of diabetic nephropathy. Regression analyses found a strong association between increasing urine levels of periostin correlated with declining GFR and increasing albuminuria independently of the degree of blood pressure, glycemic and lipid levels in patients with type 2 diabetes. Data from previous studies confirmed that renal periostin expression was strongly related to the progression of experimental hypertensive nephropathy and experimental model of CKD from 5/6 nephrectomy, ureteric obstruction and streptozotocin-induced diabetes. [8,12] Increased urine periostin levels also correlated directly with higher proteinuria and impaired renal function among progressive proteinuric and nonproteinuric CKD patients and CAN patients. [8,10] Therefore, the appearance of urine periostin underscores its value as a potential common biomarker for CKD including diabetic nephropathy.

A limitation of this research was the cross-sectional study design of increased urinary periostin with different stages of diabetic nephropathy. Further research should focus on a large population using a prospective cohort study for the role of urinary periostin in prognostic biomarkers to monitor the progression and therapeutic control of diabetic nephropathy. In addition, further studies are required to examine the pathogenic mechanisms of elevated periostin levels and their role in the early diagnosis of diabetic nephropathy. Unfortunately, we did not demonstrate periostin expression in renal tissue from normoalbuminuria and microalbuminuria stages of diabetic nephropathy. However, previously, we could detect an increment in periostin expression by immunohistochemistry and immunoblotting method in early stages of experimental models of diabetic nephropathy. Finally, although urinary periostin increased in normoalbuminuric type 2 diabetes compared with healthy controls, mean age and estimated GFR differed between both groups. Aging process with averaged GFR at 70 mL/min/1.73 m^2^ in normoalbuminuric type 2 diabetes might explain the higher levels of urine periostin. It was consistent with the results of regression analyses that found a strong association between increasing urine periostin levels correlated with advanced age, and decline of estimated GFR.

In conclusion, the results of the study identify periostin as a novel renal biomarker associated with albuminuria and GFR levels in type 2 diabetes patients. The study has also demonstrated that urinary periostin levels increased before the onset of microalbuminuria and that urinary periostin can be an early biomarker of renal tubular injury in normoalbuminuric patients with type 2 diabetes compared with controls. Measuring periostin in the urine may provide an earlier diagnosis and advanced interventions in type 2 diabetes patients.

## Supporting Information

S1 Dataset Patient data. Data from urine periostin measurements.
(XLS)
